# Acquisition of multi voxel size X-ray computed tomography and optical microscopy image datasets of a thermoplastic CFRP tape

**DOI:** 10.1016/j.dib.2026.112571

**Published:** 2026-02-09

**Authors:** Benedikt Boos, Silvia Gomarasca, Ran Tao, Christoph Queck, S.M. Amin Hosseini, Clemens Dransfeld, Martin Gurka

**Affiliations:** a Leibniz Institute of Composite Materials GmbH, Erwin-Schroedinger-Str. 58, 67663 Kaiserslautern, Germany; b Delft University of Technology, Department of Aerospace Structures and Materials, Kluyverweg 1, 2629HS, Delft, Netherlands

**Keywords:** X-Ray CT, Microscopy, CFRP, Thermoplastic, Composite, Tape, Multi Voxel Size

## Abstract

Four specimens were prepared from one continuous Carbon Fiber Reinforced Thermoplastic Polymer (CFRP) tape and nondestructively tested using 2D X-ray micrographs and 3D X-ray Computed Tomography (CT). They were each polished on one front side and imaged by optical microscopy using a Keyence VK-X1000 confocal scanning microscope. These two-dimensional micrographs provided high-resolution reference data of the polished tape surfaces. CT was performed on the same specimens with a Zeiss Xradia 520 Versa at voxel sizes of 0.8, 2.0, and 3.5 µm each. The field of view was adjusted to include the polished front side, and the rotation axis was kept constant in between scans of one specimen. This configuration enabled the CT datasets to be registered into a common coordinate system. The registered stacks were subsequently cropped to the tape volume to optimize memory usage. The 3D CT datasets were segmented using structure tensor analysis and Trainable Weka Segmentation to extract fiber, matrix and pore regions in the CFRP tapes’ microstructure. The 2D microscopy images were used as complementary benchmarks to evaluate the required spatial resolution. The overall aim was to determine whether reliable microstructural characterization demands full fiber-level resolution, or whether coarser CT scans provide sufficient information.

Specifications Table

**Table:** 

Subject	Engineering & Materials science
Specific subject area	X-Ray CT Region of Interest Scans of 4 CFRP Tape Specimen with three different voxel-sizes each. Segmentation results of individual fibers, matrix and pores using structure tensor analysis and Trainable Weka Segmentation. High-resolution microscopy images of one edge of each specimen.
Type of data	Raw Images (optical microscopy, X-ray CT Projections), Segmented Images (X-ray CT reconstructions)
Data collection	Image Acquisition (X-ray and microscopy) - Laboratory X-ray CT: Zeiss Xradia 520 Versa - Optical Microscopy: Keyence VK-X1000 confocal scanning microscope Image Processing Algorithms: - Structure Tensor Analysis [1] - Trainable Weka Segmentation [2]
Data source location	Delft (Netherlands)
Data accessibility	Repository name: Multi voxel size X-Ray Computed Tomography and Optical Microscopy Image Datasets of a Thermoplastic CFRP Tape Data identification number: 10.4121/3a864c60-3023-45ab-a6c6-f36a23d67f56 Direct URL to data: https://data.4tu.nl/datasets/3a864c60-3023-45ab-a6c6-f36a23d67f56
Related research article	The data sets presented in this paper were used in [3] where the feasibility of single fiber tracking and structure tensor analysis at the different voxel-sizes was investigated.

## 1 Value of the Data

• Overlapping regions of interests (ROI) across different voxel sizes can be used for development and assessment of quantitative image analysis algorithms such as e.g. fiber volume or pore content distribution, or general morphology quantification, for benchmarking of structure descriptors at different length scales and training ROI identification across datasets.
• Nested scan measurements support multi-scale quantitative analysis of structural feature distribution and propagation in unidirectional fiber reinforced composites.
• The included raw data (X-ray projections) supports the development or benchmarking of Computed Tomography reconstruction algorithms or associated processing pipelines.
• Since both the raw data and all analysis steps are available for three different voxel sizes, the data set is also suitable for the development or validation of novel algorithms to increase resolution (e.g. super resolution).
• Segmented Datasets also serve as a benchmark for fiber/pore volume estimation, fiber orientation analysis.
• Segmented Datasets can be used for FE model creation.
• Variety of data of containing the same microstructural information of similar specimens makes it valuable for broader range of research applications.

## 2 Background

Quantitative description of composite microstructure at various length scales, ranging from single fiber diameter to specimen size (µm to cm) is the prerequisite for advanced modeling and simulation of processing or material properties and for quality control of continuously reinforced thermoplastic composites. Due to the large range on the length scale, suitable processing pipelines for data reduction along the length scale must be developed, which include all steps from imaging (e.g. using X-ray CT including reconstruction), over image processing to evaluation with quantitative descriptors.

The provided dataset can be used as a benchmark for such developments as it contains the raw data and key analysis results of four specimens cut from a unidirectional carbon fiber reinforced thermoplastic polymer (CFRP) tape for three independently measured magnifications, resp. three different voxel sizes. This way the data contains also all realistic artifacts from imaging and stochastic noise, which are not affected by down sampling of high-resolution data.

The provided dataset covers the workflow from the acquisition of X-ray CT projections, reconstructed CT volume data to post-processing at three different voxel-sizes. The goal is to use this data set to develop new methods for a quantification of the three-dimensional microstructure of such CFRP tapes at various length scales. Staggered voxel sizes will help identify the most suitable microstructural parameters for this purpose. This is because the choice of voxel size in X-ray CT evaluations determines which structures can be distinguished from one another and influences the field of view. Microscopy images of the front sides of the four test specimens were taken to determine if microstructural parameters could also be derived from a 2D micrograph.

## 3 Data Description

**Fig. 1:**
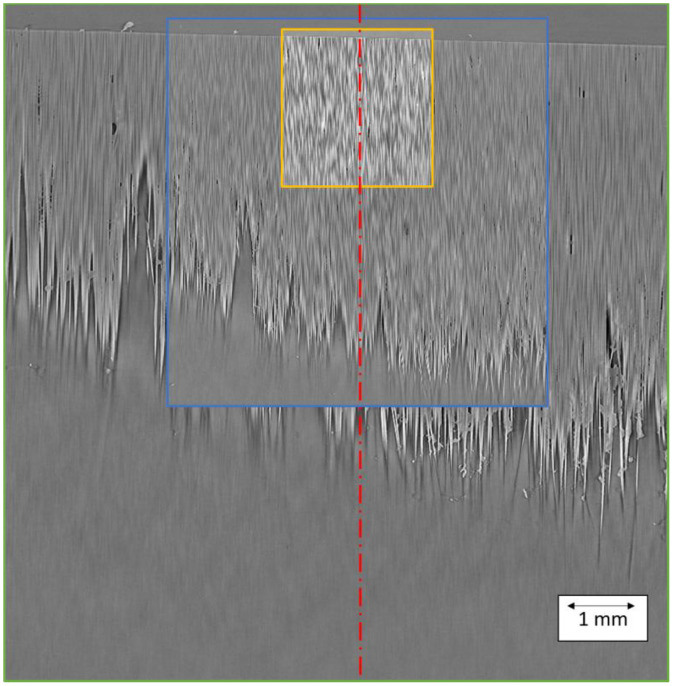
In-plane slice overview of the relative positions of the individual scans of the same specimen sharing the same axis of rotation (red). The colored frames correspond to the field of view for the different voxel sizes (orange: 0.8 µm, blue: 2.0 µm, green: 3.5 µm). The images were taken from specimen 4.

The image stacks described in this article were generated through X-ray CT scanning with reconstructed voxel sizes of 0.8 µm, 2.0 µm and 3.5 µm of four CFRP tape specimens, optical microscopy imaging of a polished cross section as a reference and subsequent quantitative processing. One edge of each specimen was polished before microscopy images were acquired of these edges. The fields of view (FOV) for the X-ray CT scans were chosen in such a way, that the polished edge was visible, but the axis of rotation remained the same for each specimen (see Fig. 1).

**Fig. 2:**
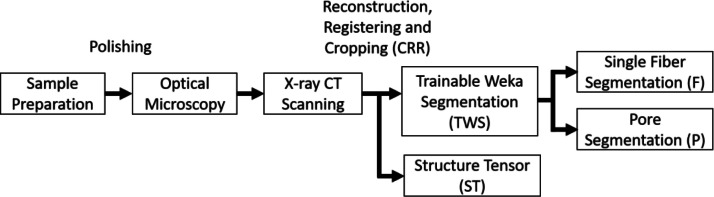
Overview of the data generation workflow.

After the X-ray CT scan acquisition, the projection datasets for each specimen and voxel size were reconstructed using Zeiss’ reconstruction software [4]. In a next step, the reconstructed images were cropped and registered for each specimen. Finally, these datasets were processed using the structure tensor analysis [1] and the Trainable Weka Segmentation [2] (see Table 1). The metadata to each dataset is provided in form of .json and ReadMe files. An overview of the entire workflow can be found in Fig. 2.

**Table 1:** Overview of the provided image datasets for each of the four specimens.

Description	Voxel/Pixel Size [µm]	Filename	Datatype
Microscopy Images	0.7	[*specimenID*]_Xn_Ym	.vk4, .png
Reconstructed Image Dataset	0.8	[*specimenID*]_0.8um	.txm
	2.0	[*specimenID*]_2.0um	.txm
	3.5	[*specimenID*]_3.5um	.txm
Cropped and Registered Reconstructed (CRR) Image Stack	0.8	[*specimenID*]_0.8um_CRR	.tif(f)
	2.0	[*specimenID*]_2.0um_CRR	.tif
	3.5	[*specimenID*]_3.5um_CRR	.tif
Structure Tensor (ST) Processed CRR Image Stack	0.8	[*specimenID*]_0.8um_CRR_ST	.mat
	2.0	[*specimenID*]_2.0um_CRR_ST	.mat
	3.5	[*specimenID*]_3.5um_CRR_ST	.mat
Trainable Weka Segmentation (TWS) Processed CRR Image	0.8	[*specimenID*]_0.8um_CRR_TWS_F	.tif
Stack (Fiber segmentation) (F)	2.0	[*specimenID*]_2.0um_CRR_TWS_F	.tif
Trainable Weka Segmentation (TWS) Processed CRR Image	0.8	[*specimenID*]_0.8um_CRR_TWS_P	.tif
Stack (Pore segmentation) (P)	2.0	[*specimenID*]_2.0um_CRR_TWS_P	.tif
	3.5	[*specimenID*]_3.5um_CRR_TWS_P	.tif

## 4 Experimental Design, Materials and Methods

### 4.1 Materials

The material used was a unidirectional tape, Toray Cetex 1225, with a fiber areal weight of 145 g/m^2^, commercially produced by Toray Advanced Composites [5]. The tape consists of an LM-PAEK matrix reinforced with standard modulus carbon fibers. From the available 30 cm-wide tape, four 2.5 cm × 2.5 cm specimens were cut in the direction transverse to the fiber alignment using a Gerber automated cutting machine equipped with a knife cutter featuring a 6 mm blade lead, operating at a cutting speed of 100 cm/s.

**Fig. 3:**
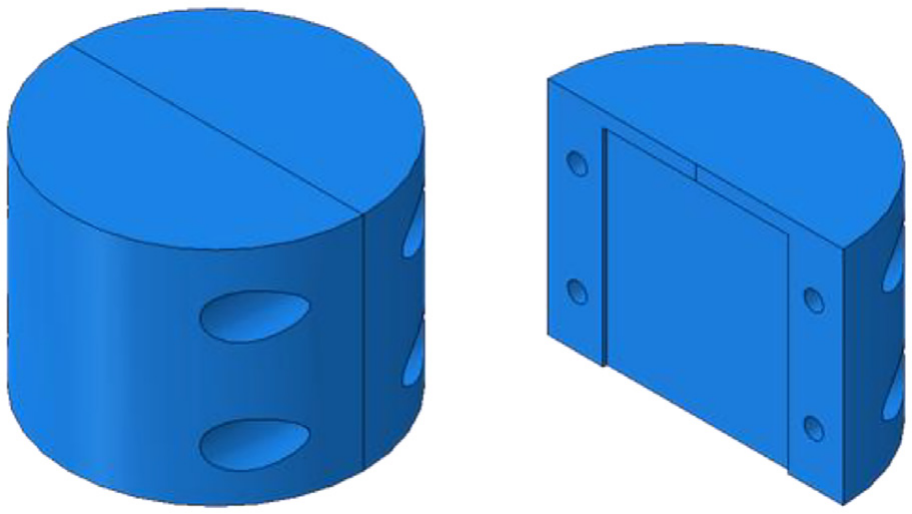
Schematic of the specimen holder for tape grinding and polishing prior to microscopy.

To enable optical microscopy, the four extracted specimens were ground and polished after being placed in a custom-designed, disassemblable specimen holder, which allowed for subsequent removal of the specimens for XCT measurements (see Fig. 3. The design included four screw connections to secure the two halves in place, and a groove to accommodate the specimen. The specimen holder was manufactured with a Formlabs Form 3+ printer using their propriety Clear Resin formulation. Polishing was performed with a Struers Tegramin-20 Grinding and polishing machine, according to the steps listed in Table 2.

**Table 2:** Steps of the polishing process.

Polishing step	Duration[s]	Force [N]	Disk rotation speed [rpm]	Holder rotation speed [rpm]	Rotation direction	Polishing agent
SiC Foil #320	20	25	300	150	Co-rotation	Water
SiC Foil #1000	30	25				
SiC Foil #2000	60	20				
SiC Foil #4000	120	15				
MD-DUR 3	240	25	150		Counter-rotation	Diamond solution
MD-DUR 1	120	20				
chem OP-S	120	10				

### 4.2 Optical microscopy acquisition

Optical microscopy was conducted via a Keyence VK-X1000 confocal scanning microscope with a 20x lens, leading to images with a pixel size of 0.7 µm. After microscopy, the specimens have been removed from the 3D printed specimen holder to be scanned by X-ray CT.

### 4.3 X-ray CT scan acquisition

**Fig. 4:**
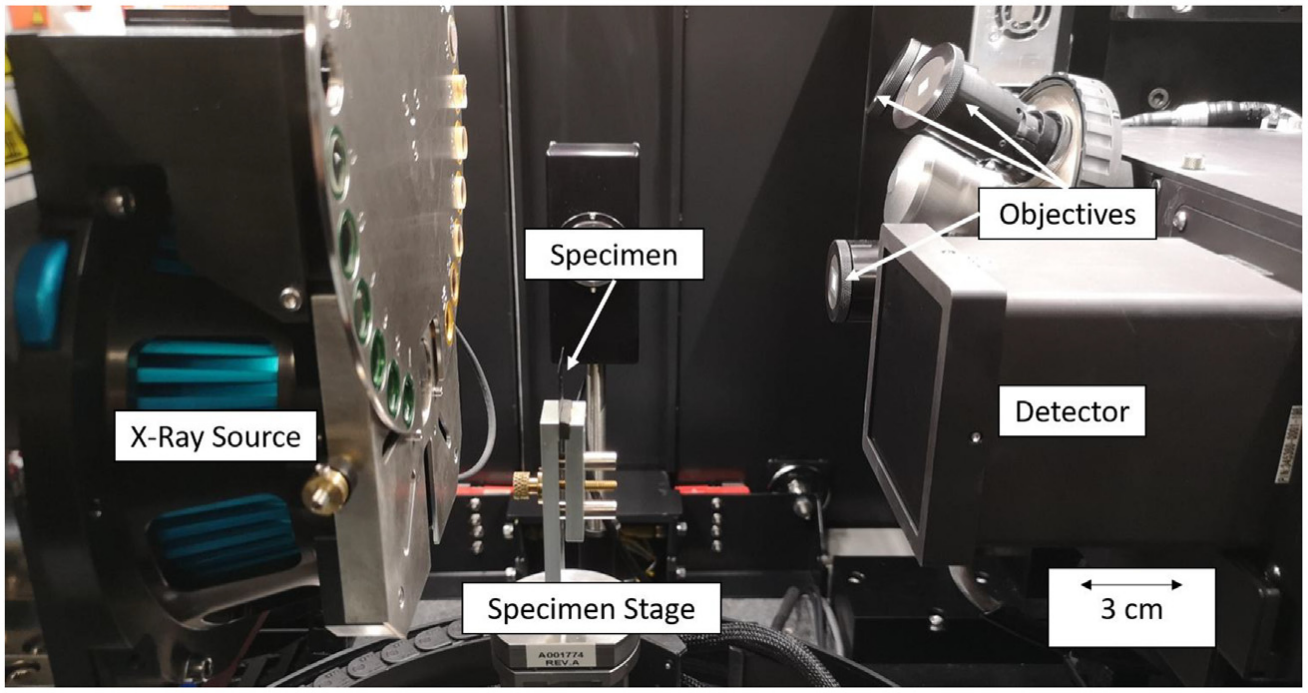
Scanning Setup in Zeiss Xradia 520 Versa.

The individual scans were acquired using a laboratory X-ray CT (Zeiss Xradia 520 Versa, see Fig. 4) combining absorption and phase contrast, with 800 nm, 2 µm and 3.5 µm voxel size. All scans were performed with an acceleration voltage of 80 kV, an electrical power of 7 W and without additional beam filtering. For the 800 nm and 2 µm voxel size scans, the x4 optical magnification and for the 3.5 µm voxel size, the 0.4x optical magnification was used in front of the detector to acquire the region of interest (ROI) scans. The CCD detector with 2024×2024 pixels without pixel binning was used. This resulted in FOVs of ∼1.6 × 1.6, 4.0 × 4.0 and 7.0 × 7.0 mm^2^ respectively. A total of 2001 projections were acquired for a full rotation of 360° for each scan. The exposure time had to be adjusted for all four scans: 12.6 s for the 800 nm scan, 5.0 s for the 2 µm scan and 8.0 s for the 3.5 µm scan. After each scan, the projections were reconstructed using the FDK algorithm [6], which is provided by the Zeiss Scout and Scan software (Version 16) associated with the CT [4]. In addition, this software was used to optimize the image quality, as it offers motion and beam hardening as well as ring artifact correction.

The specimen was clamped in the specimen holder with the polished side facing upwards. The FOVs were selected so that this upper edge, which was imaged via optical microscopy, was visible also in every X-ray CT scan (see Fig. 1). For this purpose, the specimen position was only adjusted in height, but the coordinates of the rotation axis were not changed between the scans.

### 4.4 Volume registration

**Fig. 5:**
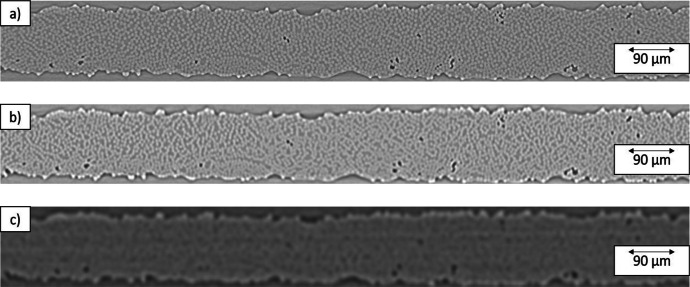
Single slice view of the relative positions in different scans: a) 800 nm voxel size, b) 2 µm voxel size, c) 3.5 µm voxel size. The images are taken from specimen 1.

Volume registration was performed in Fiji by manually rotating each dataset to align it with the principal orientation of the specimen and to ensure that the tape appeared horizontal (see Fig. 5). In all cases, the required angular corrections were <4° as reported in Table 3.

**Table 3:** Angles of rotation used for registering the three reference planes for the four Specimens analyzed.

Specimen ID	Rotation on XY plane in °	Rotation on ZY plane in °	Rotation on XZ plane in °
1	−0.9	2.1	0
2	0.6	−3.7	1
3	−0.8	2.2	0
4	0.4	1.6	1.23

Rotations were applied with respect to three reference planes:

• XY plane: transverse cross-section of the specimen;
• XZ plane: aligned with the fiber direction and the tape width;
• YZ plane: aligned with the fiber direction and the tape thickness.

The rotation procedure followed these steps:

1. The dataset was first rotated within the XY plane
2. It was then rotated 90° to the left and resliced to enable rotation within the YZ plane
3. Finally, it was resliced again, rotated back by 90°, and resliced once more to perform the rotation within the XZ plane.

Reslicing was performed with an ‘Avoid interpolation’ setting.

From the registered volumes, ROIs of overlap between the hierarchically nested volumes have been extracted for each Specimen.

Due to the low thickness of the tape, the majority of the reconstructed volumes consisted of air. For this reason, the ROIs were cropped to the area of the tapes (see Fig. 5).

### 4.5 Trainable Weka segmentation

Segmentation was performed on the ROIs extracted from the X-ray Computed Tomography volumes using Trainable Weka Segmentation (TWS) [2,7]. The algorithm enables trainable multiclass classification. In this study, a classifier was trained to identify fibers in the 0.8 µm and 2 µm datasets, while pore and air space identification was carried out across all datasets. It was not possible to perform a single fiber segmentation in scans with a voxel size of 3.5 µm because the resolution was not high enough to distinguish individual fibers. Within TWS’ settings, the training features Gaussian blur, Sobel filter, Hessian, Difference of Gaussians and Membrane Projections with a membrane thickness of 1, a membrane patch size of 19, a minimum sigma of 1.0 and a maximum sigma of 19 were selected. The FastRandomForest option was chosen as classifier.

### 4.6 Structure tensor analysis

Structure tensor (ST) analysis was used to capture local fiber orientations based on an open-source Python-based scheme [1]. Apart from several technical revisions to better adapt the scheme to our datasets, the main modification was to apply the ST analysis directly to the raw data without a gray scale Otsu-threshold filtering. Instead, after the local orientation was obtained at each voxel through the ST analysis, the original results (named unfiltered) were filtered using the segmentation masking. Both the unfiltered and masked analysis results were recorded.

The analyses were carried out on the extracted ROIs for three different voxel sizes: 0.8 µm, 2 µm, and 3.5 µm. The values of smoothing scale (σ) and integration scale (ρ) were selected according to the voxel size via the relations proposed in the original Python scheme, with resulting values as detailed in Table 4.

**Table 4:** Selected values of the parameters used for the ST analysis calculation and post-processing: smoothing scale (σ) in voxel, integration scale (ρ) in voxel, and anisotropy threshold value (β).

Dataset	0.8 µm	2 µm	3.5 µm
Unfiltered	σ =3.09, ρ =12.36	σ =1.24, ρ =4.96	σ =0.71, ρ =2.84
Segmentation masking	σ =3.09, ρ =12.36	σ =1.24, ρ =4.96	σ =0.71, ρ =2.84

## Limitations

• During transport of the test specimens, debris accumulated on the polished surface, as visible from Fig. 1 as brighter regions above the specimen’s free surface. Further post-processing steps are therefore necessary for correct feature segmentation of the X-ray CT scans and microscopy images.
• Phase contrast measurement in the Zeiss Xradia 520 Versa results in pronounced phase fringes in image areas with material phase boundaries with significantly different densities (matrix/fibers to air), which can make segmentation and post-processing difficult.

## Ethics Statement

The authors confirm that they have read and followed the ethical requirements for publication in Data in Brief and confirming that the current work does not involve human subjects, animal experiments, or any data collected from social media platforms.

## Credit Author Statement

**Benedikt Boos:** Investigation (X-ray CT)**,** Visualization, Writing - Original Draft*.* **Silvia Gomarasca:** Resources**,** Formal analysis, Visualization, Writing - Original Draft. **Ran Tao:** Resources, Formal analysis, Writing - Review & Editing. **Christoph Queck:** Investigation (X-ray CT), Writing - Review & Editing*.* **S.M. Amin Hosseini:** Investigation (Microscopy)*,* Writing - Review & Editing. **Clemens Dransfeld:** Supervision, Writing - Review & Editing. **Martin Gurka:** Supervision, Writing - Review & Editing. **All**: Conceptualization

## Data Availability

4TU.ResearchDataMulti-Voxelsize X-Ray Computed Tomography and Optical Microscopy Image Datasets of a Thermoplastic CFRP Tape (Original data)
